# Supplementary figures and images for: Adenomatous polyposis coli-binding protein end-binding 1 promotes hepatocellular carcinoma growth and metastasis
Source: PLoS One. 2020 Sep 21;15(9):e0239462. doi: 10.1371/journal.pone.0239462 (PMC7505586; doi:10.1371/journal.pone.0239462)

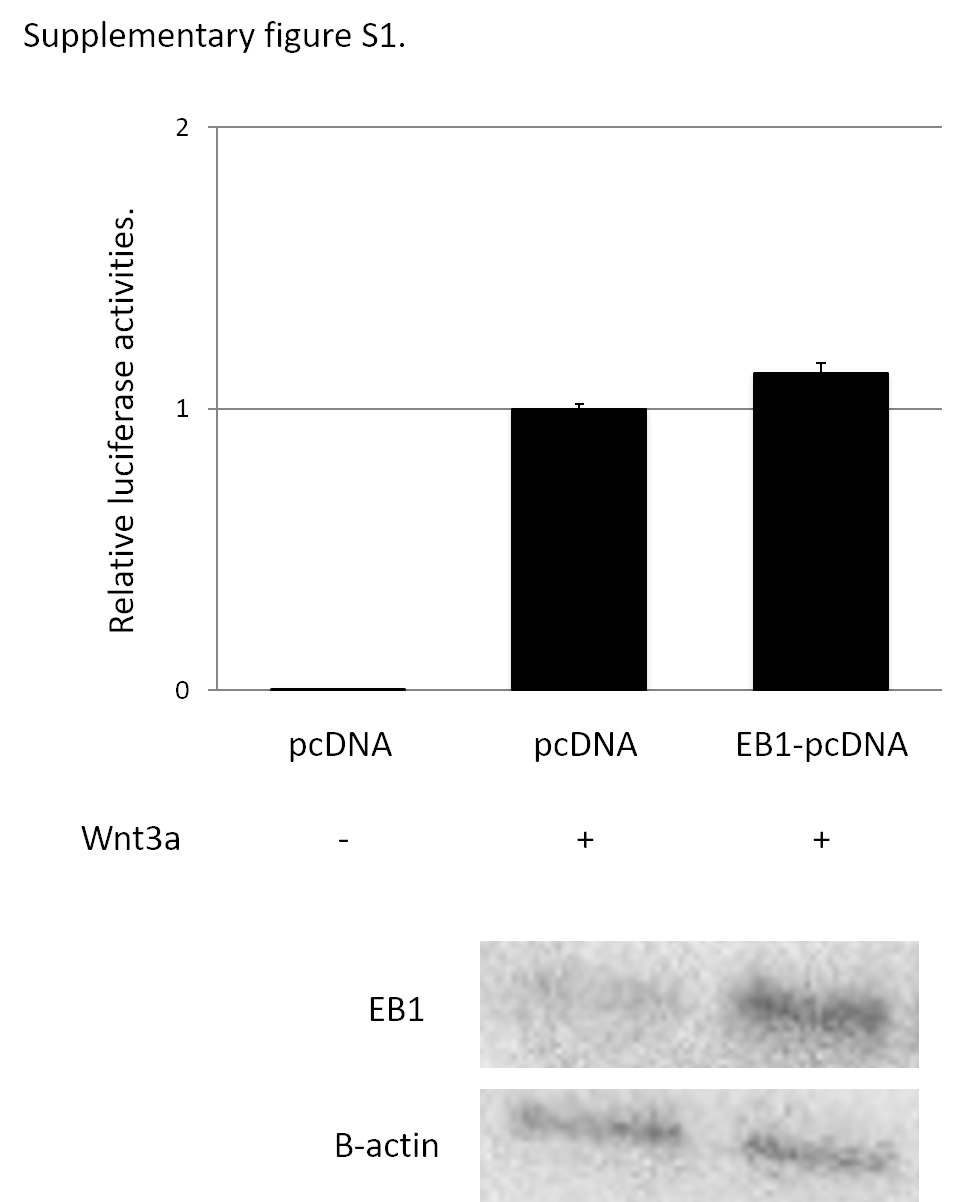

Supplement: S1 Fig — SuperTopFlash 293 reporter cells containing the αβ-catenin/Tcf reporter system 19 were transfected with empty or EB1 expression pcDNA3.1 plasmid, stimulated with Wnt3a-conditioned medium, and measured for their luciferase activities. Although EB1 elevation was detected at the protein level, there was no difference in luciferase activities in the cells transfected with the empty or EB1 expression plasmid. (TIF) [file pone.0239462.s002.tif]

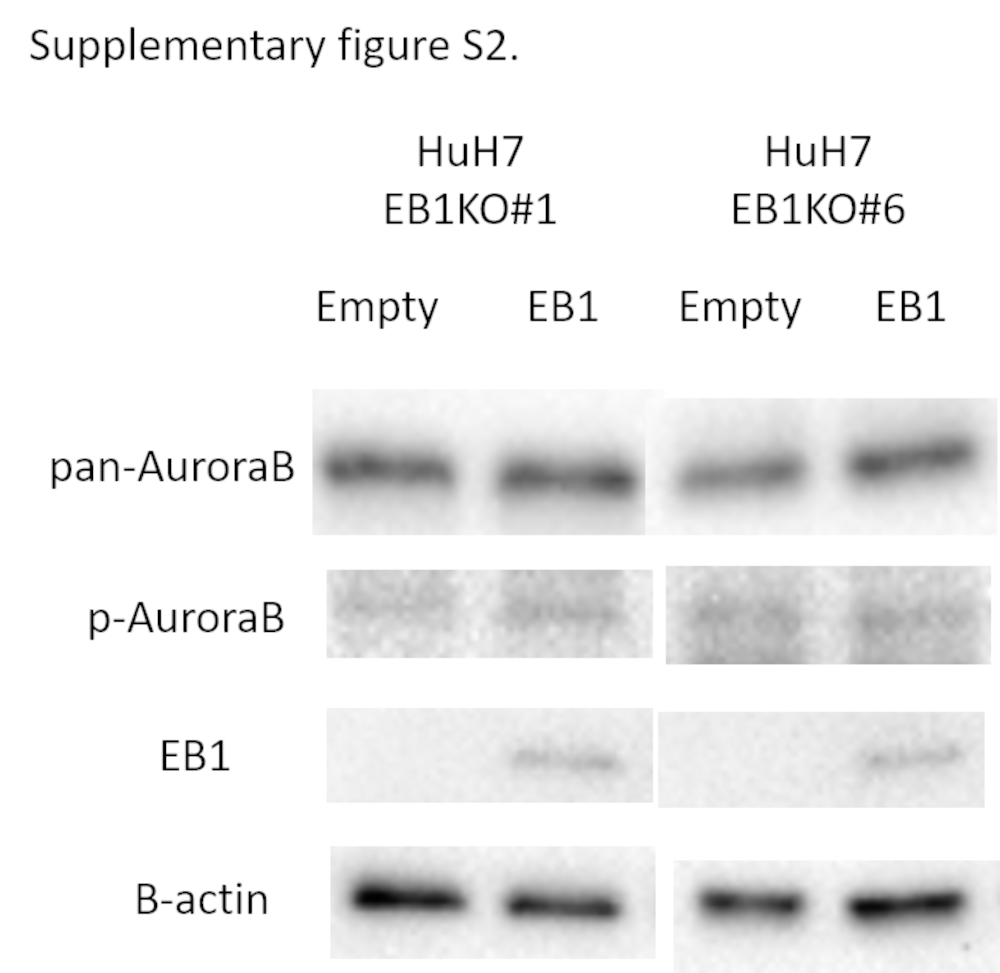

Supplement: S2 Fig — There was no significant activation of aurora-B kinase in EB1-KO HuH7 cells re-expressing EB1 compared with EB1-KO HuH7 cells. (TIF) [file pone.0239462.s003.tif]

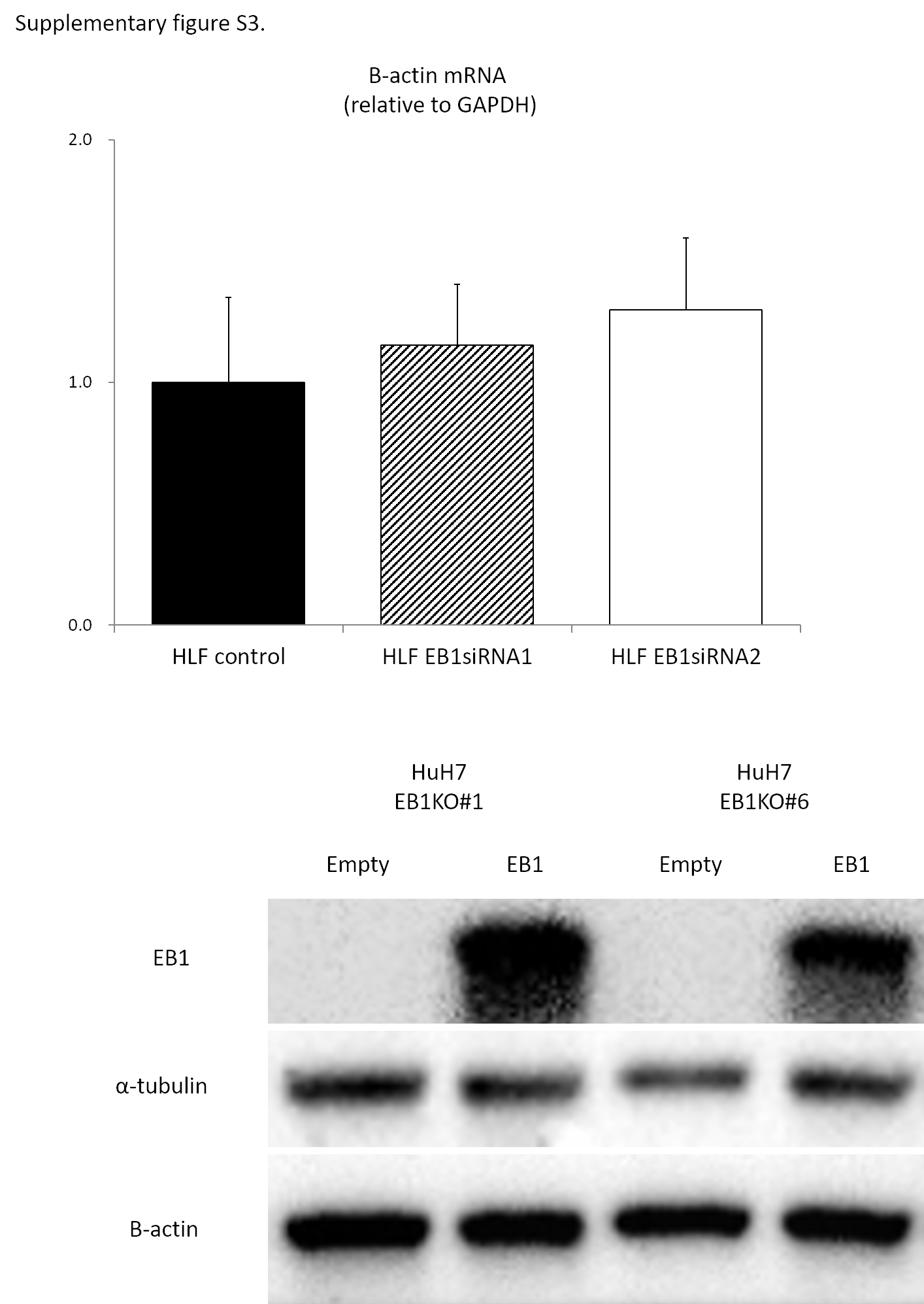

Supplement: S3 Fig — (TIF) [file pone.0239462.s004.tif]

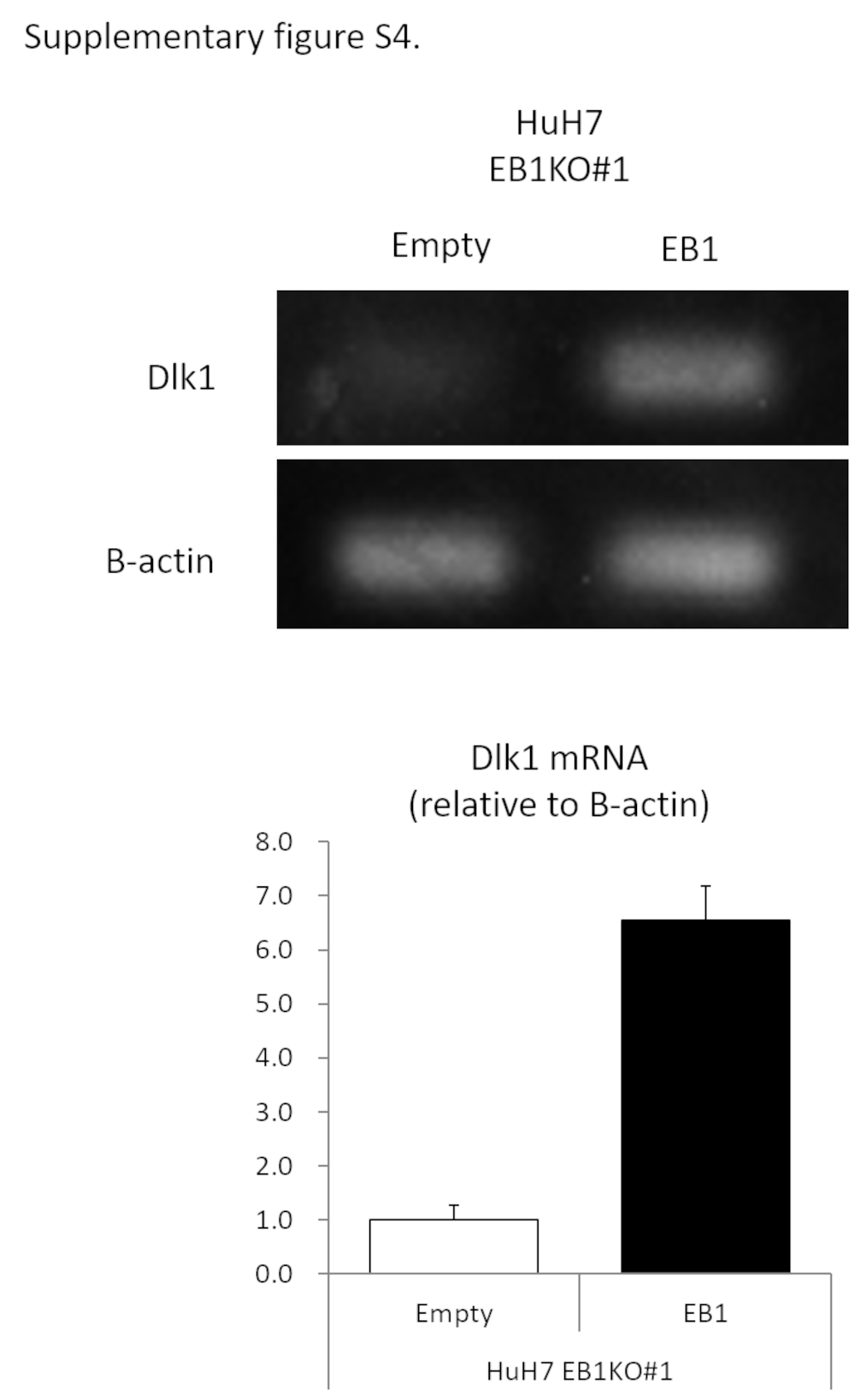

Supplement: S4 Fig — (TIF) [file pone.0239462.s005.tif]

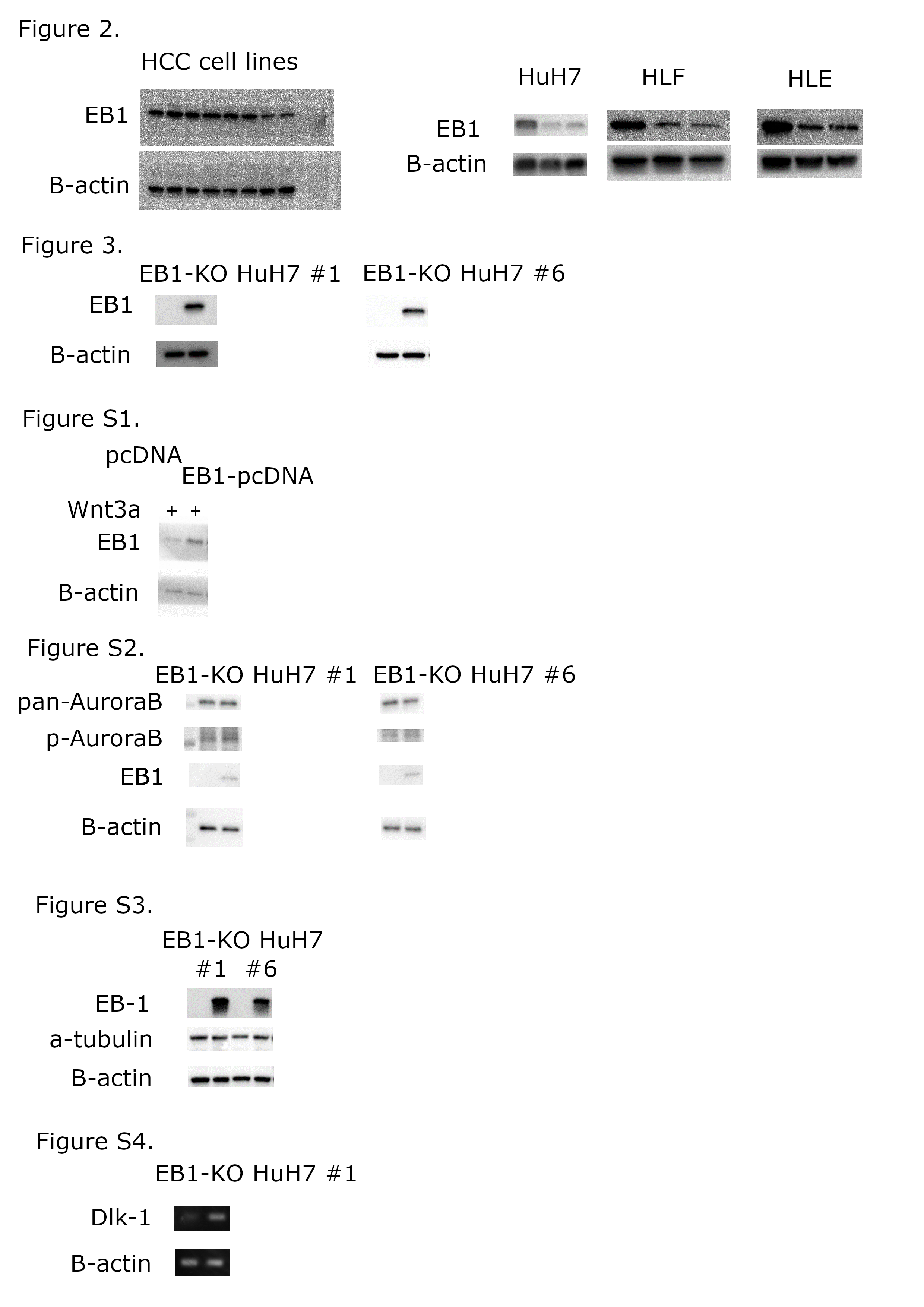

Supplement: S1 Raw images — (TIF) [file pone.0239462.s006.tif]
