# Supplementary material for: Adenomatous polyposis coli-binding protein end-binding 1 promotes hepatocellular carcinoma growth and metastasis
Source: PLoS One. 2020 Sep 21;15(9):e0239462. doi: 10.1371/journal.pone.0239462 (PMC7505586; doi:10.1371/journal.pone.0239462)
Supplement: S1 Table — (DOCX) [file pone.0239462.s007.docx]

**S1 Table. Primers**

| Name |  | Sequence |
| --- | --- | --- |
| EB1 | Forward | 5’- CCTGGATCAATGAGTCTCTGC-3' |
|  | Reverse | 5’- CACTTTCTTCAAGGCAATGGA-3' |
| β-actin | Forward | 5’- CTGTGGCATCCACGAAACTA-3' |
|  | Reverse | 5’- GTACTTGCGCTCAGGAGGAG-3' |
